# Sexual Dimorphism of Cardiovascular Ischemia Susceptibility Is Mediated by Heme Oxygenase

**DOI:** 10.1155/2013/521563

**Published:** 2013-09-17

**Authors:** Anikó Pósa, Krisztina Kupai, Rudolf Ménesi, Zita Szalai, Renáta Szabó, Zoltán Pintér, György Pálfi, Mariann Gyöngyösi, Anikó Berkó, Imre Pávó, Csaba Varga

**Affiliations:** ^1^Department of Physiology, Anatomy and Neuroscience, Faculty of Science and Informatics, University of Szeged, Közép Fasor 52, Szeged H 6726, Hungary; ^2^Department of Biological Anthropology, University of Szeged, Közép Fasor 52, Szeged H 6726, Hungary; ^3^Department of Cardiology, Medical University of Vienna, Waehringer Guertel 18-20, A-1090 Vienna, Austria

## Abstract

We investigated the gender differences in heme-oxygenase (HO) enzyme, which produces endogenous vascular protective carbon monoxide (CO). We studied (1) the activity and expression of HO enzymes in the left ventricle (LV) and aorta, (2) basal increase in basal blood pressure provoked by arginine vasopressine (AVP) *in vivo*, (3) the heart perfusion induced by AVP, (4) the ST segment depression provoked by adrenaline and 30 seconds later phentolamine, and (5) the aorta ring contraction induced by AVP in female and male Wistar rats. We found that HO activity and the expression of HO-1 and HO-2 were increased in female rat aorta and LV. We demonstrated that the basal blood pressure and administration of AVP provoked blood pressure response are increased in the males; the female myocardium was less sensitive towards angina. Both differences could be aggravated by the inhibition of HO. The aorta rings were more susceptible towards vasoconstriction by AVP in males; isolated heart perfusion decrease was higher in males. The HO inhibition aggravated the heart perfusion in both sexes. In conclusion, the increased HO activity and expression in females might play a role in the sexual dimorphism of cardiovascular ischemia susceptibility during the reproductive age.

## 1. Introduction

Gender-based differences in the incidence of hypertensive and coronary artery disease, the development of atherosclerosis, and myocardial remodelling after infarction are attributable to the direct effect of oestrogen on the myocardium, vascular smooth muscle (VSM), and endothelium. Cardiovascular morbidity and mortality are far less in premenopausal women compared to age-matched men, but the basis of this discrepancy remains controversial. Ovarian hormones are believed to be mainly responsible for this “female advantages” in cardiovascular function although the underlying mechanism has not been fully elucidated.

In the heart and vasculature oestrogen mediates rapid vasodilation via production of nitric oxide (NO), protects against neointimal injury in the balloon-injured rat and facilitates the re-endothelialization of the damaged vessel [1], reduces both myocardial infarct size and occurrence of ischemia- and reperfusion-induced ventricular arrhythmias in canine heart [2].

Carbon monoxide (CO) is a product of heme oxygenase (HO) as well and is not an antioxidant but can cause induction of antioxidant genes [3–5]; it also decreases superoxide (O_2_^−^) levels [6, 7], increases gluthione (GSH) levels [8], and has an antiapoptotic effect [9, 10]. Further, CO is a vasodilator, which has been shown to play an important role in the regulation of basal and constrictor-induced vascular tone, in blood vessels [11, 12].

Almost all CO produced *in vivo* comes from the degradation of heme by HO. CO and NO have similar properties; both behave as messenger signalling molecules, are able to induce the relaxation of blood vessels through vasodilation and inhibit the proliferation of vascular smooth muscle cells (VSMC) [13]. Like NO, HO-derived CO influences the soluble guanylyl cyclase (sGC) and cGMP pathways, which serve to regulate both blood pressure and vascular contractility [14]. It has been demonstrated that by upregulating the HO system in young (8-week-old) spontaneously hypertensive rats, coincidently, sGC and cGMP levels rise, which leads to a significant reduction of blood pressure. On the other hand, by using an inhibitor of HO-1 activity, the blood pressure of rats undergoing HO-1 inhibition significantly increases [14, 15]. HO is the rate-limiting enzyme in heme catabolism; it catalyzes the breakdown of heme into equimolar amounts of carbon monoxide, biliverdin, and free iron [16]. Three mammalian HO isoforms have been identified, one of which, HO-1, is a stress-responsive protein induced by a remarkably vast panoply of stimuli [16–19]. Mounting evidence indicates that HO-1 plays an important cytoprotective role [20–23]. This enzyme has been found to have beneficial effects in a wide variety of pathological conditions, such as inflammation, atherosclerosis, and ischemia/reperfusion injury [20–22, 24, 25]. In noncardiac tissues, there is evidence that HO-1 is regulated by NO [26, 27]. Of the metabolites generated by HO-1 catalysis, biliverdin (and bilirubin) has been shown to possess antioxidant activity, whereas carbon monoxide has been found to exert many salutary effects in various settings, including myocardial ischemia [16, 28, 29].

The aim of the present work was to investigate any gender-based differences in HO expression and activity and to clarify the role of HO enzyme system in cardiovascular protection via using HO enzyme system inhibitor tin protoporphyrin IX.

## 2. Materials and Methods

### 2.1. Examined Groups

We used male and female Wistar rats (230–250 g) bred in our animal house; the breeding stock was derived from the Laboratory Animals Producing Institute (Gödöllő, Hungary). Each group consisted of at least ten animals. Rats were housed in a light-controlled room under constant environmental conditions and fed pellet rat chow and tap water ad libitum after they were received in our laboratory. The 12 : 12 h light-dark cycle started at 6:00 AM, and the room temperature was maintained at 20–23°C. All OVX rats were in the proestrus stage, which is characterized by the unique presence of nucleated epithelial cells stained with a 0.1% Giemsa solution and observed under light microscopy (×100) [30].

**Figure 1:**
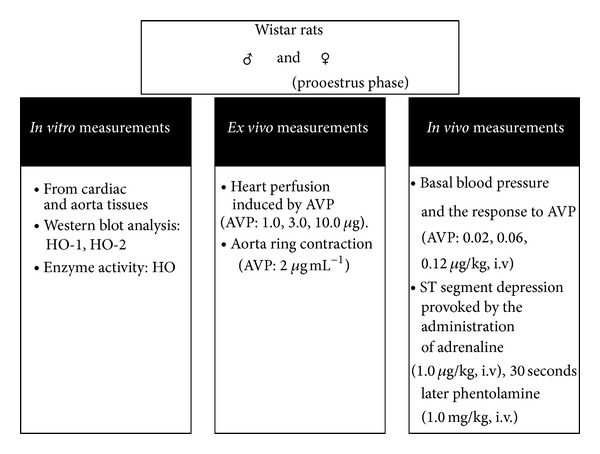
Experimental design: *in vitro*, *ex vivo*, *in vivo* measurements from heart aorta in intact female (in the proestrus phase) and male Wistar rats HO-1: heme oxygenase 1; HO-2: heme oxygenase 2; AVP: Arginine vasopressin.

Heme-oxygenase enzymes were inhibited by tin protoporphyrine IX (SnPP; 30.0 mg/kg, s.c., pH 7.4, 24 hours and one hour before treatment). Experimental design is shown in Figure 1. All manipulations were performed in accordance with the standards of the European Community guideline on the care, and use of laboratory animals and had been approved by the Institutional Ethics Committee.

### 2.2. Cardiac and Aortic HO-2 and HO-1 Protein Expressions

The expression of HO-2 and HO-1 enzymes was determined by Western blot analysis. Cardiac and aorta tissues were homogenized (Ultra Turrax T25; 13.500 min-1; 2 × 30 s) in ice-cold Tris-mannitol buffer (2.0 mM Tris 7–9, 50.0 mM mannitol, 100.0 *μ*M phenyl-methyl-sulphonyl-fluoride, 2.0 *μ*M leupeptin, 0.50 mU/mL aprotinin, 0.50% Triton X-100) and were centrifuged for 20 min at 12000 g at 4°C. Protein content was measured by spectrophotometric assay (Bio-Rad Protein Assay).

Aliquots of 25.0 *μ*g of total cellular protein were denatured by mixing and boiling with 20.0 mM Tris 7–9, 3.0 mM EDTA, 2.0% sodium dodecyl sulphate (SDS), 10.0% *β*-mercaptoethanol, and 20.0% glycerol. The samples were electrophoresed (100 V, 50 mA) on 10.0% polyacrylamide gel and transferred (100 V, 100 mA, 2 h) to nitrocellulose membrane (Amersham, Pharmacia Biotech., Buckinghamshire, UK). Equal protein loading was determined by staining the blot with 0.10% Ponceau red in 5.0% acetic acid. Two hours after blocking with PBS (pH 7.4), 0.25% tween 20, and 5.0% fat-free dried milk, the membrane was probed with mouse anti-HO-1 monoclonal antibody (1/10.000; 2 h) (StressGen Biotechnologies Corp., Victoria, Canada) or anti-HO-2 monoclonal antibody (1/1000; 2 h) (StressGen Biotechnologies Corp., Victoria, Canada) at room temperature, washed 3 times with PBS-tween 20 and then incubated with horseradish peroxidase-conjugated bovine antimouse antibody (1/2000; 1 h; Santa Cruz Biotechnology Inc., Santa Cruz, Ca, USA) for 1 h at room temperature. Membranes (Hybond ECL Nitrocellulose membrane, Amersham, Pharmacia Biotech., Buckinghamshire, UK) were developed by using an enhanced chemiluminescence system (ECL+Plus, Amersham Pharmacia Biotech., Buckinghamshire, UK) and exposed to Hyperfilm (Biomax light-1, Eastman Kodak Comp. Rochester, New York). Films were analysed by using the ImageQuant Software (Amersham Pharmacia Biotech., Buckinghamshire, UK) after scanning with GelAnalyst 3.01 Software (Iconix, Toronto, Canada). Results are expressed as %, and the 100% is the maximal expression.

### 2.3. Cardiac and Aorta HO Enzyme Activities

The cardiac left ventricle and aortic tissues were homogenised (Ultra Turrax T25; 13.500 min-1; 2 × 30 s) in ice-cold 10.0 mM N-2-hydroxyethylpiperazine-N′-2-ethanesulfonic acid (HEPES), 32.0 mM sucrose, 1.0 mM dithiothreitol (DTT), 0.10 mM EDTA, 10.0 *μ*g/mL trypsin inhibitor, 10.0 *μ*g/mL leupeptin, and 2.0 *μ*g/mL aprotinin (pH: 7.4). The supernatant was collected by centrifugation for 20 min at 15000 g at 4°C. The reaction mixture contained the following compounds in a final volume of 1.50 mL: 2.0 mM glucose-6-phosphate, 0.14 U/mL glucose-6-phosphate dehydrogenase, 15.0 *μ*M hemin, 120.0 *μ*g/mL rat liver cytosol as a source of biliverdin reductase, 2.0 mM MgCl_2_ × 6H_2_O, 100.0 mM KH_2_PO_4_, and 150.0 *μ*L of supernatant. To start the reaction 100.0 *μ*L of *β*-nicotinamide adenine dinucleotide phosphate, reduced form (*β*-NADPH; 150.0 *μ*M) was added to the samples; then they were incubated in darkness at 37°C for 60 min. The reaction was stopped by placing the samples on ice. Bilirubin solution was used as standard (58.47 *μ*g/mL; 10.0 *μ*M). The bilirubin formed was calculated from the difference between optical densities obtained at 464 and 530 nm. Protein content was determined by spectrophotometric assay (Bio-Rad Protein Assay).

One unit of HO activity was defined as the amount of bilirubin (nmol) produced per hour per mg protein.

### 2.4. Measuring of Basal Blood Pressure and the Response of Blood Pressure to AVP

Animals were anaesthetized with 30.0% urethane (0.50 mL/100 g, i.p.) and then pretreated with phentolamine (10.0 mg/kg, i.p). A single bolus injection of arginine vasopressin (AVP; 0.02; 0.06; 0.12 *μ*g/kg, i.v.) was administered into the tail vein following the stabilisation of blood pressure. The procedure has been described in detail previously [31]. Briefly, the elevation of blood pressure (expressed as the percentage of maximal increase as compared to the basal value) was measured in the right carotid artery through a blood pressure transducer connected to the HAEMOSYS computerised complex haemodynamic analysis system (Experimetria UK, London). The core temperature of rats was maintained at 37°C with a homeothermic control unit (Harvard Instrument, UK).

### 2.5. Measuring of Heart Perfusion According to Langendorff

Animals received an intraperitoneal injection of heparin (500 units) 10–20 min before being euthanized. After cervical dislocation, hearts were rapidly excised (mean time to perfusion 2 min) and mounted on a Langendorff perfusion system. Hearts were perfused via the aorta according to the Langendorff method at a constant pressure of 70.0 Hgmm at 37°C. The perfusion medium was a Krebs-Henseleit buffer consisting of 118.0 mM NaCl, 4.70 mM KCl, 2.50 mM CaCl_2_, 1.18 mM MgSO_4_, 25.0 mM NaHCO_3_, 1.18 mM KH_2_PO_4_, and 5.50 mM glucose. The perfusate was bubbled with a 95% O_2_/5% CO_2_ through a glass oxygenator and adjusted to pH 7.4. After a stabilization period of 15 min, the heart perfusion was measured (expressed as the percentage of maximal response as compared to the basal value) as a response to AVP (AVP final concentration in Krebs solution: 1.0; 3.0; 10.0 *μ*g).

### 2.6. Experimental Angina Provoked by Epinephrine Plus Phentolamine

The standard limb lead II of the surface electrocardiogram (ECG) was recorded by the HAEMOSYS system. The change in ST segment was measured and used as the index of angina severity. The mean ECG voltage 13 ms after the peak of the S wave was defined as the value of the ST segment, as described previously [32]. The difference in the amplitude of the ST segment after and before the administration of angina-provoking agents was calculated and expressed as the depression of the ST segment in mV. In the epinephrine plus phentolamine model, a single dose of epinephrine (10.0 *μ*g/kg) and 30 s later *α*-adrenoceptor antagonist phentolamine (15.0 mg/kg) were administered into the tail vein of the rat. Each agent was dissolved in 0.20 mL of physiological saline and injected over 2 s. The ECG, heart rate, and blood pressure changes were recorded simultaneously.

### 2.7. Measurement of Surviving Aorta Contraction

The rats were killed by cervical dislocation, and the abdominal aortas were removed and placed in chilled Krebs-Henseleit bicarbonate solution (4°C), which was gassed with 95% O_2_ and 5% CO_2_. The composition of the incubation solution was described in detail by [33]. The aortas were cleaned of all adipose and connective tissue, the abdominal region was cut into rings (3 mm long), and their weights were measured. Two adjacent aortic rings were studied from each animal in paired fashion. The rings were mounted on two 25-gauge stainless steel wires; the lower one was attached to a stationary stainless steel rod and the upper one to a force-displacement transducer for the measurement of isometric tension. The transducer was connected to an ISOSYS computerized programme system (Experimetria, UK, London) for continuous recording of the blood vessel tension.

Immediately after being mounted, the abdominal rings were suspended in water-jacketed organ baths filled with 15.0 mL of incubation solution maintained at 37°C and continuously gassed with 95% O_2_ and 5% CO_2_. Before the start of the experiments, the blood vessels were gradually stretched (over a 30 min period) to an optimum passive tension of 2.50 g and equilibrated for a period of 20–30 min. Following the equilibration period, we freshly added the same dose of arginine vasopressin (2 *μ*g mL^−1^) to the incubation solution. The optimum contractile response to vasopressin was calculated before the experiments by using gradually increasing vasopressin doses. The contractile response to vasopressin was expressed in terms of the tension of the aorta ring (g/mg ring weight).

### 2.8. Chemicals

The following chemicals were ordered from different companies: Arginine-vasopressine (AVP; Organon, The Netherlands), Urethane (Reanal, Hungary), Phentolamine (Regitin, P; Ciba-Geigy, Switzerland) and tin protoporphyrine IX (SnPP; Frontier Scientific Europe, United Kingdom). All compounds not specified above were derived from Sigma Aldrich.

## 3. Results

### 3.1. HO-2 and HO-1 Expression of Cardiac Left Ventricle and Aorta 

**Figure 2:**
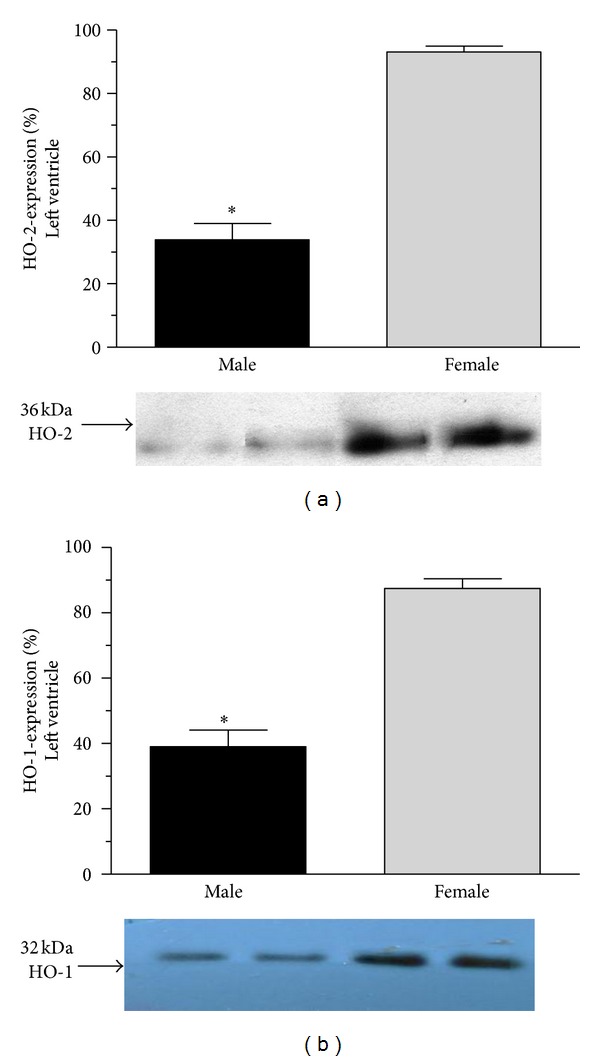
Heme-oxygenase expression (HO-2 and HO-1 expressed as %) in the cardiac left ventricle of male (the black square) and female (the grey square). Data are expressed as means ± S.E.M. of the results of a minimum of 10 rats per group. Statistical significance: *P < 0.001. Panel (a): heme-oxygenase 2 (HO-2) (expressed as %) in the cardiac left ventricle tissue of male (the black square) and female (the grey square) rats with densitometric assessment (means ± S.E.M. expressed as %, 100% is the maximal expression). Panel (b) shows heme-oxygenase 1 (HO-1) (expressed as %) in the left ventricle tissue of male (the black square) and female (the grey square) rats with densitometric assessment (means ± S.E.M. expressed as %, 100% is the maximal expression). Data are expressed as means ± S.E.M. of the results of a minimum of 10 rats per group. Statistical significance: *P < 0.001 as compared to the female group.

**Figure 3:**
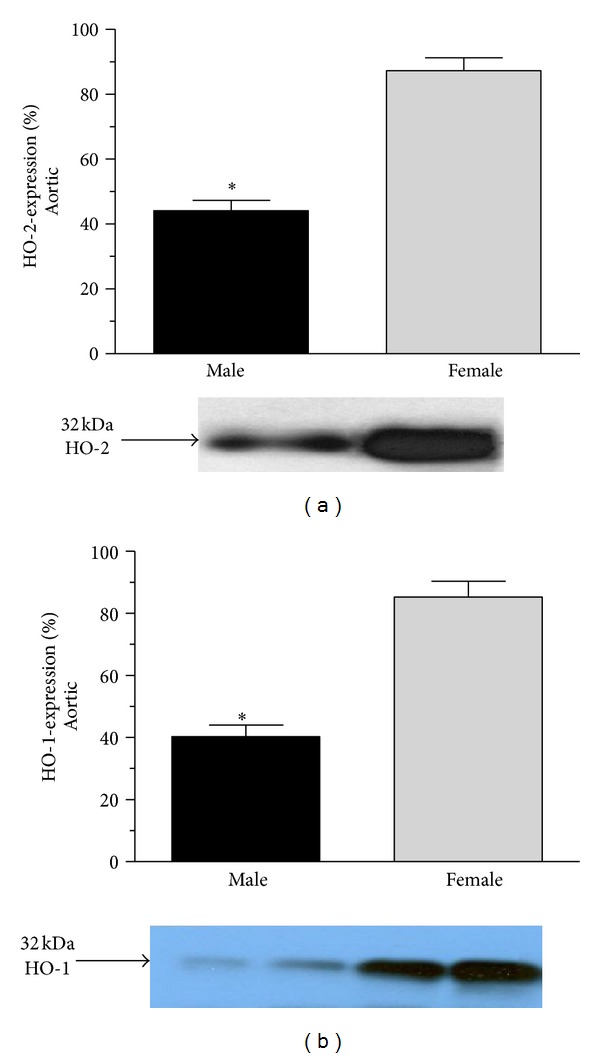
Heme-oxygenase expression (HO2 and HO-1 expressed as %) in the aortic of male (the black square) and female (the grey square). Data are expressed as means ± S.E.M. of the results of a minimum of 10 rats per group. Statistical significance: *P < 0.001. Panel (a): heme-oxygenase 2 (HO-2) (expressed as %) in the aortic tissue of male (the black square) and female (the grey square) rats with densitometric assessment (means ± S.E.M. expressed as %, 100% is the maximal expression). Panel (b) shows heme-oxygenase 1 (HO-1) (expressed as %) in the aortic tissue of male (the black square) and female (The grey square) rats with densitometric assessment (means ± S.E.M. expressed as %, 100% is the maximal expression). Data are expressed as means ± S.E.M. of the results of a minimum of 10 rats per group. Statistical significance: *P < 0.001 as compared to the female group.

HO-2 and HO-1 protein was determined by Western-blot techniques. Significantly (*P* < 0.001) decreased cardiac HO enzymes expression was found in males left ventricle (HO-2: 33.857 ± 5.161%; HO-1: 39.0 ± 5.113%) and in aorta (HO-2: 44.143 ± 3.112%; HO-1: 40.286 ± 3.790%) as compared to the females left ventricle (HO-2: 93.143 ± 1.792%; HO-1: 87.429 ± 3.015%) and aorta (HO-2: 87.286 ± 4.028%; HO-1: 85.286 ± 5.126%). Data are shown in Figures 2 and 3.

### 3.2. HO Activity of Cardiac Left Ventricle and Aorta

**Figure 4:**
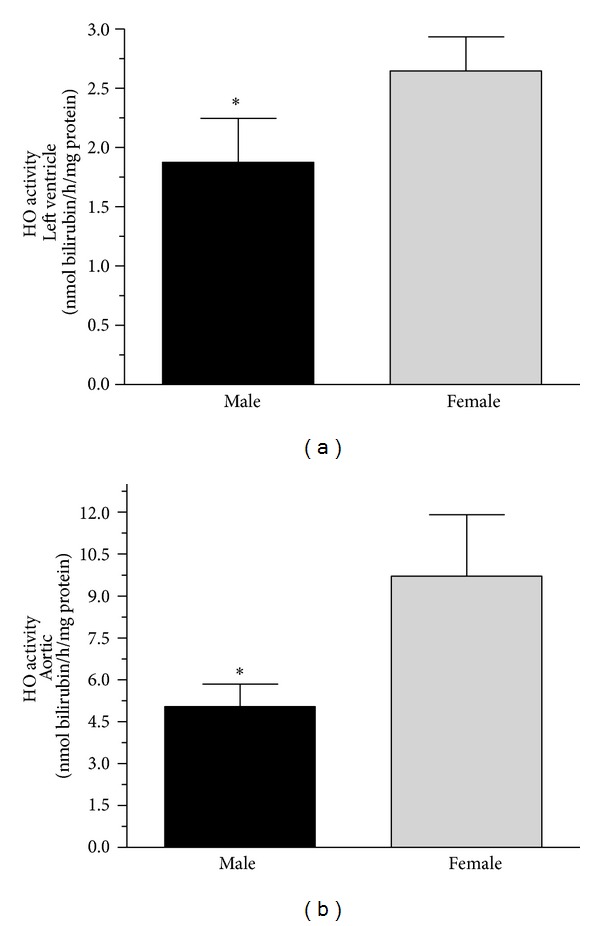
Heme-oxygenase activity (HO; expressed as nmol bilirubin/h/mg protein) in the cardiac left ventricle (a) and aorta tissues (b) of male (the black square) and female (the grey square). Data are expressed as means ± S.E.M. of the results of a minimum of 10 rats per group. Statistical significance: *P < 0.05.

HO-enzyme activity was determined by measurement of bilirubin formation. In male group, activity of HO was significantly (*P* < 0.05) decreased both in the cardiac left ventricle (1.877 ± 0.369 nmol bilirubin/h/mg protein) and aorta (5.045 ± 0.798 nmol bilirubin/h/mg protein) HO enzyme activity decased as compared to the females cardiac left ventricle (2.647 ± 0.288 nmol bilirubin/h/mg protein) and aorta (9.709 ± 2.201 nmol bilirubin/h/mg protein). Data are shown in Figure 4.

### 3.3. The Measuring of Basal Blood Pressure and the Effect of HO Inhibition on Blood Pressure as a Response to AVP

**Figure 5:**
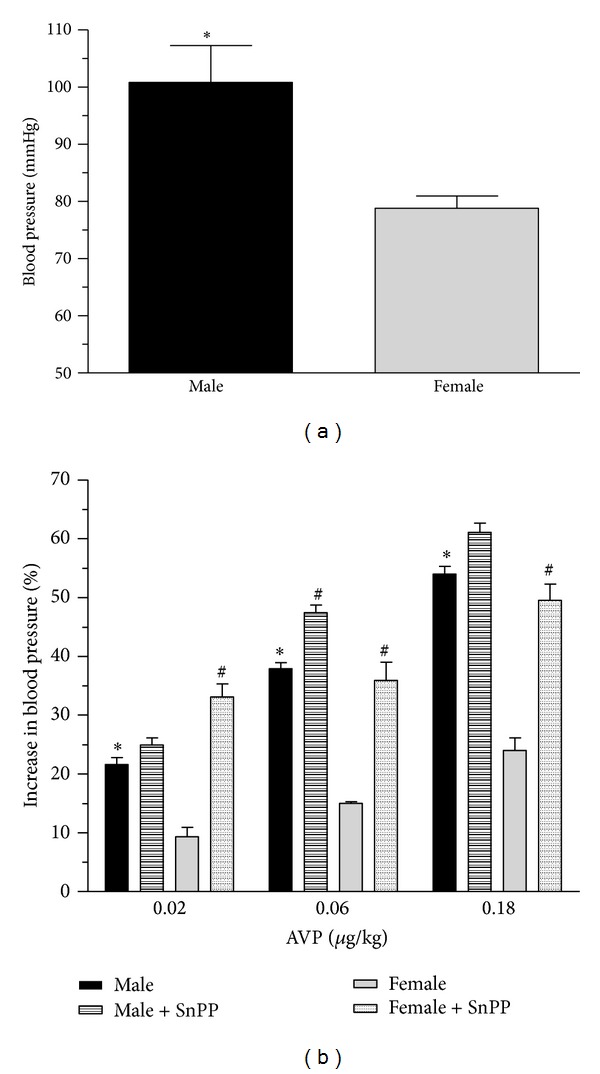
Panel (a) shows basal blood pressure (mmHg) and Panel (b) shows the effect of HO inhibition by tin protoporphyrin IX (SnPP: 30.0 mg/kg, pretreatment 24 hours and one hour prior to the measurement) on the increase in arterial blood pressure after administration of arginine vasopressin (AVP; 0.02, 0.06, 0.18 *μ*g/kg) in male (the black square) and female (the grey square) rats measurement. Results are shown as means ± S.E.M. for 10 animals in each group. Statistical significance: *P < 0.05 as compared to the ovary intact group and ^#^ *P* < 0.05 a significant difference between groups with and without SnPP pretreatment.

The basic blood pressure is shown in Figure 5(a). Significantly (*P* < 0.05) higher blood pressure was measured in the control male rats as compared to the females (100.80 ± 6.49 versus 78.80 ± 2.19 mmHg).

The arterial blood pressure was measured in the right carotid artery and we demonstrated the increase in blood pressure induced by intravenous administration of AVP (0.02-0.18 *μ*g/kg) in catecholamine-depleted (phentolamine: P, 10.0 mg/kg i.p.) female and male rats. 

AVP caused a dose-dependent increase in arterial blood pressure both in the female and male rats. In the females (9.30 ± 1.62–24.0 ± 2.12%), AVP induced a significantly (*P* < 0.05) lower elevation in blood pressure than in males (21.60 ± 1.19–54.0 ± 1.26%). The inhibition of HO enzyme system caused significant augmentation in all groups (female: 31.1 ± 2.23–49.5 ± 2.76%; male group: 24.90 ± 1.12–61.10 ± 1.53%). Data are shown in Figure 5(b).

### 3.4. The Effect of Inhibition of HO on Isolated Heart Perfusion as a Response to AVP

**Figure 6:**
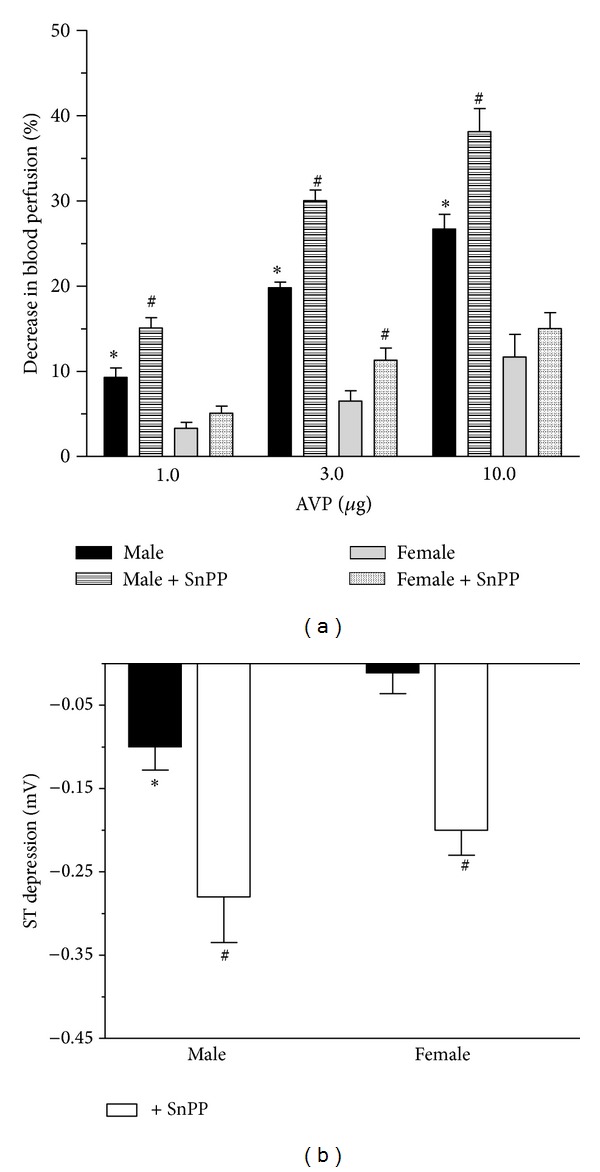
The diagrams show the effect of HO inhibition by tin protoporphyrin (SnPP) on the decrease in heart perfusion (a) expressed as the percentage of change, measured by incubation liquid given AVP (1.0, 3.0, 10.0 *μ*g) in hearts of male (the black square) and female (the grey square). The Panel (b) demonstrates effect of the HO inhibitor tin protoporphyrin (SnPP) on ST segment changes (measured in a lead II standard surface ECG; expressed in mV) following intravenous injection of epinephrine (10.0 *μ*g/kg) and 30 s later phentolamine (15.0 mg/kg). White columns (the white square) show the intact groups without SnPP treatment. Patterned columns show the actions of SnPP (30.0 mg/kg pretreatment 24 hours and one hour prior to the measurement). Results are shown as means ± S.E.M. for 10 animals in each group. Statistical significance: *P < 0.05 as compared to the ovary intact group and ^#^ *P* < 0.05 a significant difference between groups with and without SnPP pretreatment.

The perfusion was measured according to Langendorff. The effect of AVP (1.0–10.0 *μ*g) on heart perfusion proved to be dose dependent in all groups. In the male animals (9.30 ± 1.108–26.70 ± 1.711%), AVP caused a significantly (*P* < 0.05) higher decrease of heart perfusion than in intact female group (3.30 ± 0.72–11.70 ± 2.61%). The inhibition of HO enzyme system caused (SnPP, 30.0 mg/kg, pretreatment 24 hours and one hour prior to the measurement) significant augmentation in all groups (female: 5.10 ± 0.83–15.0 ± 1.90%; male group: 15.10 ± 1.19–38.10 ± 2.72%). Data are shown in Figure 6(a).

### 3.5. The Effect of Inhibition of HO on ST Depression

ST segment changes were measured in a lead II standard surface ECG following intravenous injection of epinephrine (A: 10.0 *μ*g/kg) and 30 s later phentolamine (P: 15.0 mg/kg) in female and male rats. The administration of phentolamine 30 s after epinephrine caused a significant (*P* < 0.05) ST segment depression only in male group (−0.10 ± 0.0278 mV). In females, an ST segment depression did not develop. Pretreatment with SnPP (30.0 mg/kg, pretreatment 24 hours and one hour prior to the measurement) caused ST depression in female (−0.20 ± 0.03 mV) and augmented the ST depression in males (ST segment change: −0.28 ± 0.055 mV). Data are shown in Figure 6(b).

### 3.6. The Measurement of Aorta Contraction Provoked by AVP

**Figure 7:**
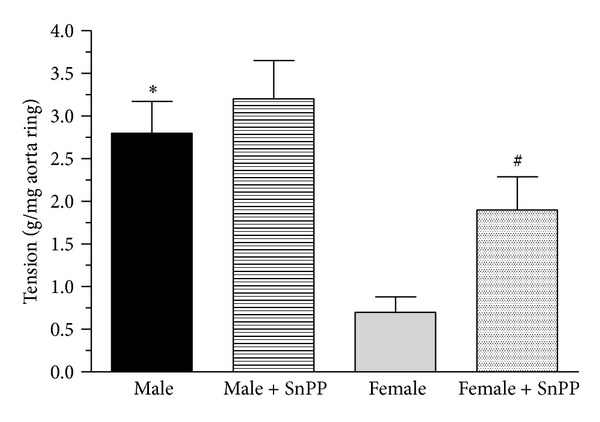
The diagrams show the effect of HO inhibition by tin protoporphyrin (SnPP) on the aortic contraction expressed as g/mg ring weight, measured by incubation liquid given AVP (2.0 *μ*g/mL) in male (the black square) and female (the grey square) rats. Patterned columns show the actions of SnPP (30.0 mg/kg) pretreatment (24 hours and one hour prior to the measurement). Results are shown as means** **±** **S.E.M. for 10 animals in each group. Statistical significance: *P < 0.05 as compared to the ovary intact group and ^#^ *P* < 0.05 a significant difference between groups with and without SnPP pretreatment.

Results observed in the experiment involving the surviving aorta ring contraction are demonstrated in Figure 3(b). The vasoconstriction induced by AVP was much significantly (*P* < 0.05) higher in the male than that observed in the females (2.80 ± 0.37 versus 0.70 ± 0.18 g/mg aorta ring). The inhibition of HO enzyme system caused (SnPP, 30.0 mg/kg, pretreatment 24 hours and one hour prior to the measurement) augmentation in all groups (male: 3.20 ± 0.45 g/mg aorta ring female group: 1.90 ± 0.39). Data are shown in Figure 7.

## 4. Discussion

The present study revealed gender difference in vascular and myocardial HO expression, and activity, which may contribute to the gender-related difference of cardiac function. The current experiments are reported, that are gender differences in the basal blood pressure, basal aorta contraction, the experimental angina, and heart perfusion. This notion of “female advantage” is supported by the gender-related differences in the clinical manifestations of cardiovascular disease such as stroke, LV hypertrophy, and coronary heart disease.

Several hypotheses have been postulated for the gender-related difference of cardiovascular morbidity and mortality including differences in hormones, lipid profile, myocardial, endothelial, and vascular performance between male and female genders and aging [34–36]. While a great part of the observed gender differences in vascular reactivity has been attributed to genomic modulation by sex hormones, nongenomic effects of those hormones also exist. In fact, relatively little is known about the HO activity changing during aging in the heart. Lavrovsky et al. [37] found enhanced oxidative stress during aging which is accompanied by compensatory induction of the antioxidant enzyme HO-1 through activation of the NFkB pathway in the rat liver. Ariyoshi et al. [38] examined the effects of age and sex on microsomal heme oxygenase activity and cytochrome P-450 content in rat liver. They observed that heme oxygenase activity declined with an increase in age, namely, its activity in 100 days old (young) rats was 58% in male and 72% in female rats as compared with respective 30-day-old (immature) rats, and in 300-day-old (old) rats, it was 32% in male and 39% in female rats. Results of Bitar fit into the literature data; namely, testosterone treatment decreased the microsomal concentration of heme in aged rats by 37%, respectively, as compared to young values. In contrast, a marked increase in the activity of microsomal heme oxygenase was seen in these animals.

Oestrogen had generally been considered a protective factor against cardiovascular disease, which was based on epidemiological data showing that the incidence of coronary heart disease among women was lower than among men before menopause, and this disparity decreased at postmenopause [39]. In postmenopausal women, the hormone replacement therapy was found to reduce the rate of cardiovascular disease. Moreover, many studies concerning the effects of long-term oestrogen administration on systemic haemodynamics in postmenopausal women. Choudhry et al. [40] showed that oestrogen administration upregulates HO activity following traumatic injury and trauma haemorrhage. The upregulated HO inhibits the expression of adhesion molecules and prevents subsequent leukocyte—endothelial cell interactions under these conditions. Moreover, upregulation of HO protects mitochondrial function and prevents ATP-depletion after oxidative stress. Liver ontogenesis of HO has been examined, and high HO activity levels were observed during fetal development and during development and aging, the transcriptional response to oxidative stress decreases, and HO-1 protein levels do not increase progressively during aging [41]. These phenomenon may be explained by a decreased transcriptional ability to respond to stress rather than by a reduction in oxidative stress [42]. Abraham and Kappas [41] found that HO-1 responds to known inducers when administered to young rats, but induction of HO-1 in old animals (24-months of age) did not change the levels of cytochrome P450 compared with the perturbations seen in young rats [43].

However, the association between oestrogen and cardiovascular disease has been corrected. It was reported that women had a worse prognosis after myocardial infarction (MI) than men did. In support of clinical observation, it was experimentally demonstrated that oestrogen replacement in ovariectomized rats resulted in an increased size of infarct after MI than placebo treatment. The authors speculated that oestrogen attenuates or downregulates a number of stress responses [44].

Rahimian et al. demonstrated a gender difference in aortic eNOS mRNA expression [45]. In accordance, Morschl et al. have reported that the cNOS activity is higher in the aorta of female rat compared to its activity of male aortic tissue [46].

In the present study, we first demonstrated gender differences in HO enzyme system, which can also play role in the cardiovascular protection and can be also upregulated by endogen oestrogen. To clarify the exact role of HO, we measured HO-2 and HO-1 protein levels and activity in the presence of HO inhibitor.

Oestrogen exerts a protective action through favourable effects on lipid profiles, decreased platelet and monocyte adhesion, and decreased vascular reactivity [47, 48]. Although the mechanisms by which oestrogen affects vascular tone are not completely understood, and a change in the communication between the vascular endothelium and smooth muscle is likely an important pathway for the action of oestrogen. The mechanisms by which gender influences CO production are unclear and may involve an increase in HO expression and activity.

Contraction of mesenteric arteries to phenylephrine was greater in arteries from male rats.

Arteries from male rats seem to be more sensitive to the modulatory effects of 17*β*-estradiol than arteries from female rats [49]. In our experiment, we found also greater contraction of abdominal aorta to AVP in male rats, and this contraction can be augmented via using HO enzyme system inhibitor.

The produced CO has been hypothesized to serve a physiological role in regulating vascular tone, which is mediated by cGMP-signalling pathway and by calcium activated potassium channels [50]. The HO system is also present and regulated in the heart. HO-1 and HO-2 differ in gene organization and structure and in chromosomal localization [51]. The two forms also vastly differ in cell type, tissue distribution, and regulation. HO-1 enzymes have been characterized as endoplasmic reticulum (ER) associated proteins, due to the abundant detection of HO activity in microsomal (104,000 g) fractions. Both HO-1 and HO-2 contain a COOH-terminal hydrophobic domain segment that suggests a general membrane compartmentalization. Recent studies have raised the possibility of the functional compartmentalization of HO-1 in other subcellular domains beside the ER, including but not limited to the nucleus and plasma membrane. The potential functional subcellular compartmentalization of HO enzymes raises an intriguing issue of organelle specific function of HO metabolites, mainly, CO [16]. Relatively little is known about the possible functional compartmentalization of HOs to the nucleus. Preliminary studies indicate that heme stimulates the nuclear translocation of HO-1. Furthermore, HO-2 was detected constitutively in the nucleus of NIH3T3 (cells mouse embryonic fibroblast cells) and was proposed to facilitate the entry of HO-1 [52]. The comparative expression of HO-1 and HO-2 in kidneys, heart, and the vasculature under normal conditions and the response to oxidative stress have recently been examined; the findings of these studies most likely have ramifications for cardiovascular system physiology. In this system, HO-2 is the predominant form expressed under normal conditions [16]. HO-2 protein is normally expressed in the endothelial and the smooth muscle layers of the blood vessels [53, 54] in the carotid body chemoreceptors [55] and in the adventitial neurons of blood vessels [53]. When stressed, there is an impressive increase in HO-1 mRNA expression in the heart, kidneys, and vasculature. In the stressed heart, HO-1 protein is expressed particularly at high levels in the arterioventricular (AV) node [56] and in the myocytes [57] where normally HO-1 protein is minimally expressed. HO-2 is reportedly absent from the striated musculature and appears to be coexpressed with NOS in vascular endothelial cells and in select nerve cell populations of certain parasympathetic and sensory ganglia [58]. Using immunostaining techniques, a widespread expression of HO-2 has been detected not only in blood vessel wall constituents (arterial and venous endothelial cells, vascular smooth muscle cells), but also in connective tissue elements (fibrocytes/fibroblasts and fibroblast-like cells). The cardiac HO system may have a role in preventing atherosclerosis, regulating blood pressure, and modulating NO-mediated myocardial preservation [59].

This experiment was designed to compare HO enzyme system activity, basal HO-2 and HO-1 expressions, and CO production in males and females in oestrus phase. The level of oestrogen in blood is increased between the morning and afternoon of proestrus and remained high during estrus morning, and it then decreases again in metestrus and diestrus. The circulating level of estradiol in the proestrus phase induces elevated expression and activity of HO enzyme system, compared to male rats [60].

The elevated expression and activity of HO enzyme system in examined females caused moderated blood pressure, aorta contraction, diminished ST depression, and normal response of blood pressure and heart perfusion provoked by AVP. HO expression and activity in heart and abdominal aorta resulting enhanced generation of basal CO. Although CO has been traditionally regarded as toxic molecule, recent evidence has revealed that this gas exerts pleiotropic homeostatic effect. In particluar, CO has been shown to promote vasorelaxation and to inhibit proliferation of vascular smooth muscle cells, apoptosis, transplant rejection, inflammation, platelet aggregation, microvascular thrombosis, cytokine production, and oxidative stress [28]. Like NO, HO-derived CO influences the sGC and cGMP pathways, which serve to regulate both blood pressure and vascular contractility. By using an inhibitor of HO activity, the blood pressure, the ST depression, and heart perfusion are significantly augmented in both female and male animals.

CO has been identified as an endogenous cellular messenger, and studies suggest an important role of CO in hemodynamic regulation [61]. It has been shown that endogenously produced CO is a signal molecule [62] and an activator of guanylyl cyclase responsible for the generation of cGMP in the vascular tissue [63]. These findings indicate that vessel wall-derived CO could serve as an endogenous regulator of vascular tone and platelet activity. HO-CO and NOS-NO pathways show many similarities; both HO and NOS have distinct constitutive and inducible isoforms, and both CO and NO stimulate sGC to produce cGMP as the second messenger effector. Moreover, many of stimuli that induce iNOS, such as catecholamines, cytokines, and ischemia/reperfusion, also induce HO-1 [64]. The relative contribution of CO and NO to the activation of GC in the cardiovascular system remains unknown, even under physiological conditions. In many pathological conditions, such as hypoxia, thermal injury, and ischemia/reperfusion, CO mediated effects may be predominant. For instance, it was found that an HO inhibitor blocked cGMP production, whereas an NOS inhibitor has no effect [65]. Results from the present study tend to implicate CO as an important cardioprotective agent in the “female advantage” in cardiovascular function. The activation of HO enzyme system in a gender-dependent manner may help explain differences observed in cardiovascular disease risk between the sexes and supports the potential beneficial effect of physiological oestrogen.
